# Value of mNGS in sonication fluid for the diagnosis of periprosthetic joint infection

**DOI:** 10.1186/s42836-019-0006-4

**Published:** 2019-09-23

**Authors:** Chongjing Zhang, Xinyu Fang, Zida Huang, Wenbo Li, Chao-fan Zhang, Bin Yang, Wenming Zhang

**Affiliations:** 1 Department of Orthopaedic Surgery, The First Affiliated Hospital of Fujian Medical University, No.20 Chazhong Road, Fuzhou, 350005 China; 2 Department of Laboratory Medicine, The First Affiliated Hospital of Fujian Medical University, No.20 Chazhong Road, Fuzhou, 350005 China

**Keywords:** Periprosthetic joint infection, Metagenomics, Next-generation sequencing, Microbiological diagnostic, Sonication fluid

## Abstract

**Objective:**

To evaluate the effectiveness of metagenomics next-generation sequencing (mNGS) for the detection of pathogenic microorganisms in periprosthetic joint infection (PJI) using the sonication fluid from removed prosthesis.

**Methods:**

In this prospective diagnostic cohort study, 44 patients who underwent revision arthroplasty in our hospital from December 2016 to December 2018 were screened. Seven cases were excluded due to incomplete clinical data, insufficient synovial fluid or failure of sequencing. According to the PJI diagnostic criteria recommended by the Musculoskeletal Infection Society (MSIS), the patients were defined as PJI or aseptic failure (AF). Conventional culture, sonication fluid culture and mNGS were performed, in order to assess the value of mNGS using sonication fluid for the diagnosis of PJI, and the mNGS results were analyzed and compared with the conventional and sonication fluid culture.

**Results:**

Among the 37 patients, 24 were diagnosed with PJI (64.86%), while 13 were diagnosed with aseptic failure. Among the 24 patients diagnosed with PJI, 15 cases (62.5%), 17 cases (70.8%) and 24 cases (100%) yielded positive results in conventional culture, sonication fluid culture and mNGS, respectively. In addition, mNGS detected the same pathogenic microorganisms in 16 out of the 17 (94.12%) culture-positive (conventional + sonication fluid) PJI cases. In the only one discrepancy case, *Enterococcus faecalis* was identified in the cultures, while *Enterobacter cloacae* was detected by mNGS. In the AF group, the results of the conventional culture were all negative. Nevertheless, *Staphylococcus epidermidis* was detected in the sonication fluid culture and mNGS in one case. The diagnostic sensitivity of mNGS for PJI was 100%, which was significantly higher than 70.83% (*P* = 0.039) of the sonication fluid culture and 62.5% (*P* = 0.021) of the conventional culture. The diagnostic specificity of mNGS for PJI was 92.31%, which was not significantly different (*P* > 0.05) from those of the conventional culture (100%) and sonication fluid culture (92.31%).

**Conclusion:**

We demonstrated that mNGS using sonication fluid can improve the detection rate of pathogenic microorganisms and provide valuable information for the diagnosis of PJI. In addition, mNGS can effectively identify pathogenic microorganisms in culture-negative PJIs cases, especially for the cases who have been treated with antibiotics before sample acquisition or have fastidious microorganisms. Therefore, this method can potentially help to guide the clinical use of antibiotics.

## Introduction

Periprosthetic joint infection (PJI) represents a serious complication after arthroplasty [1]. The key to successful treatment is timely and accurate microbial diagnosis, as the use of sensitive antibiotics is an important part of the therapeutic regimen, which can avoid unnecessary operations, prevent antibiotic abuse and improve the therapeutic effect [2]. At present, microbial culture remains the gold standard for microbial detection. However, the culture assays can be relatively insensitive, leaving 7–12% of the pathogenic microorganisms undetected in PJIs even when multiple culture media were used with prolonged culture time [3, 4]. The PJIs with fastidious microorganisms or previously treated with antimicrobials are often culture-negative, and the formation of biofilm leads to decreased number of pelagic bacteria, compromising the sensitivity of microbial detection [5].

In recent years, multiple reports have indicated that molecular diagnostics based on polymerase chain reaction (PCR) can improve the diagnostic sensitivity of PJI [6–9]. However, multiplex real-time PCR assays cannot detect certain uncommon pathogenic microorganisms due to the employment of specific primers [10]. The broad-range PCR assay allows detection of 16S rRNA gene with universal primers, but cannot identify false-positive results or detect bacteria and fungi at the same time. In addition, the broad-range PCR has poor performance in identifying polymicrobial infections [11].

Next-generation sequencing (NGS), especially the metagenomic next-generation sequencing (mNGS), is an evolving technology widely applied in clinical diagnosis [12]. mNGS combines high-throughput sequencing and bioinformatic analysis to detect microbial species and their abundances based on the BLAST database [13]. mNGS has been demonstrated to improve the microbial detection rates in oral, intracranial and pulmonary infections [14, 15]. However, the evaluation of mNGS in the diagnosis of PJI remains limited. Thoendel et al. used mNGS in a PJI case to confirm the pathogenic microorganisms, such as Mycoplasma salivariusin, in the synovial fluid [16]. Ivy et al. used mNGS to test the synovial fluid of PJIs, and identified the same pathogens in 82.9% of the culture-positive cases. In addition, the pathogens could be detected by mNGS in 84.0% of the culture-negative cases [17]. Street et al. reported a comparison of mNGS with the culture methods in 97 sonication fluid samples from PJI and other orthopedic device infections. The diagnostic sensitivity and specificity of mNGS for PJI were 88 and 88%, respectively, indicating that mNGS can provide accurate diagnostic information in PJI cases [18]. However, there are still some cases where the pathogenic microorganisms from synovial fluid and tissues fail to be detected by mNGS. The limitations may be attributed to the interference from human DNA and the biofilm formed on the surface of the prosthesis that decreases the concentration of the bacteria in the synovial fluid and tissues [19].

In recent years, sonication fluid culture from explanted prostheses has been found to improve the microbiological yield through disruption of the bacterial biofilm in PJI [20, 8]. However, this method cannot avoid the false-negative results caused by fastidious microorganisms. In addition, the bacterial membranes may become more permeable for antibiotics to penetrate, increasing the possibility for antibiotics to affect bacterial activity and reduce the positive rate of culture-based detection [21]. Importantly, mNGS is a culture-independent molecular diagnostic method that does not rely on bacterial proliferation. We hypothesize that the diagnostic efficiency can be improved by using mNGS to detect the bacterial nucleic acids in the sonication fluid from explanted prostheses. Accordingly, we conducted a prospective study that used mNGS to test the sonication fluid of the patients who underwent revision arthroplasty, and compared the results with that of culture-based method to evaluate its diagnostic efficiency.

## Materials and methods

### Inclusion and exclusion criteria

This cohort study was conducted in the First Affiliated Hospital of Fujian Medical University. The study protocols and all operations were reviewed and approved by the Ethics Committee (Ethics Number:2014[047]). Patients who underwent revision arthroplasty in our hospital from December 2016 to December 2018 were screened. The inclusion criteria were met if the patients underwent revision arthroplasty because of PJI, aseptic loosening, periprosthetic fracture. The exclusion criteria were as follows: [1] incomplete clinical information or ambiguous diagnosis of the patients, [2] insufficient synovial fluid volume, [3] specimen contamination or suspected contamination. Patients who met the standard described by the Musculoskeletal System Infection Association (MSIS) were designated as having PJI [22], and the others were diagnosed with AF. All patients were examined by a surgeon preoperatively for the presence of sinus tract in communication with the prosthesis or not. Blood routine, serum CRP and ESR were examined. All PJI patients were treated with two-stage revision, and the patients in the AF group were treated with one-stage revision.

### Sample collection

To collect the synovial fluid, joint aspiration was preoperatively performed in the patients suspected of PJI. In patients with insufficient preoperative joint fluid and patients in AF group, the joint fluid was obtained during surgery. Synovial fluid (0.5 ml) was examined for white blood cell count and neutrophil percentage. The prostheses were removed aseptically in the operation room and transported to the microbiology laboratory in a sterile and hermetic plastic box with 500 ml sterile saline solution. After 30 s of vortex oscillation, the box was placed into the water bath of an sonication cleaning machine (Oshin, Wuxi, China). The samples were sonicated at 40 kHz for 1 min, and then centrifuged for 15 min at 4000 r/min. Finally, the supernatant was discarded, and the samples were collected (1 ml synovial fluid and sonication fluid for mNGS, 1–3 ml synovial fluid and sonication fluid for microbiological culture). At least 5 periprosthetic tissue samples were collected in each case during surgery for microbiological culture.

### Conventional culture and sonication fluid culture

All samples were immediately transported to the laboratory within 30 min. Synovial fluid and sonication fluid were tested by Gram staining and acid-fast staining, followed by culturing of the bacteria and fungi on blood agar plates. The remaining synovial fluid and sonication fluid were injected into BACTEC Peds Plus/F culture bottles (Becton Dickinson, Germany) and cultured in the BACTEC 9050 Culture System (Becton Dickinson, Germany). The periprosthetic tissues were cut up and ground before staining and culturing on the blood agar plate. The cultures were incubated at 37 °C and inspected daily for 5 days (aerobic cultures) and respective 14 days (anaerobic cultures). The species in all samples were identified, and the drug sensitivity was determined using the VITEK2 System (BioMerieux, USA). The conventional cultures included synovial fluid and periprosthetic tissue cultures, and the results of sonication fluid culture were analyzed independently. Positive culture was defined as > 20 CFU/plate.

### Sonication fluid mNGS detection and analysis

The sonication fluid mNGS dectection includes the following steps: [1] Ceramic beads were utilized to break the cell wall. Total genomic DNA was extracted using the TIANamp Micro DNA Kit (DP316, Tiangen Biotech). [2] DNA libraries were constructed according to the standard protocol of the BGISEQ-500 sequencing platform (BGI-Tianjin, Tianjin, China). The quantified libraries were sequenced on the BGISEQ-500 platform. [3] The raw data from sequencing were analyzed using a bioinformatic pipeline developed by BGI. The bioinformatic analysis included the following main steps: [1] Clean reads of high-quality sequencing data were generated by filtering out the short, low-quality and low-complexity reads. [2] Human host sequences were eliminated by mapping to the human reference genome (hg19) with the Burrows-Wheeler Alignment. [3] The remaining sequencing data were aligned to the Microbial Genome Database, which contains the genomic sequences of 2,700 viruses, 1,494 bacteria, 73 fungi and 48 parasites related to human diseases. The reference genomes in the database were downloaded from the National Center for Biotechnology Information (NCBI). When the number of reads stringently mapped to pathogen species (SMRN) was below 50, the detected bacterial or fungal SMRN ≥ 3, and the SMRN was at least 5 times that of the negative-control group, the microorganism was considered pathogenic. Since the fungal nucleic acid was relatively raw, we referred to the proposal of Li et al., and set the thresholds as the relative abundance at the genus level (RAG) ≥ 30% and SMRN > 50 on the same platform. The original mNGS results included not only pathogenic microorganisms but also a large number of background microorganisms. Therefore, it was necessary to set appropriate thresholds to increase the detection rate of true pathogens and reduce the misclassification of background bacteria as pathogens. Based on the proposals in previous literature reports and our experimental results, the thresholds were set as follows: [1] Burkholderia, Ralstonia, Delftia, Sphingobium, Alternaria, Sodaria, Aspergillus, Albugo, etc. are the most common background microorganisms, which can be detected in other types of samples in the same laboratory and are rarely confirmed as pathogenic microorganisms. These microorganisms are confirmed as pathogenic microorganisms when their RAG ≥80%. [2] The microorganisms are considered meaningless when their SMRN < 3. [3] The microorganisms are considered pathogenic when they have the highest CR and SMRN. [4] *Mycobacterium tuberculosis* complex (MTCP) is recognized as a pathogen when SMRN ≥1, as its nucleic acids are extremely rare in PJI.

### Statistical analysis

Differences between two groups were evaluated using the χ^2^ or Fisher’s exact test for categorical variables. Sensitivity, specificity, positive predictive rate (PPV), negative predictive rate (NPV) and accuracy of each diagnostic method were calculated. McNemar’s chi-square test (two-side) was used to compare the sensitivity and specificity between the diagnostic tests. The SPSS 21.0 software (SPSS, USA) was used for statistical analyses.

## Results

### Demographic characteristics

The demographic data, clinical manifestations of the patients are shown in Table 1. A total of 44 cases met the inclusion criteria. According to the exclusion criteria, 3 cases with incomplete clinical data, 3 cases without adequate synovial fluid and 1 case with suspected sample contamination during sonication were excluded. A total of 37 patients (17 females and 20 males) with an average age of 65.38 ± 12.98 years (range, 30–90 years) were included. 19 had hip prostheses and 18 had knee prostheses. According to the MSIS criteria, 24 (64.86%) had chronic PJI with an average age of 67.83 ± 11.38 years (range, 46–90 years) and 13 (35.14%) had aseptic failure with a average age of 60.85 ± 14.94 years (range, 30–86 years). Within 2 weeks before sampling, the antibiotics use in the PJI group (62.5%) was significantly higher than that in the AF group (7.7%). In addition, the clinical manifestations associated with PJI, such as sinus tract communication with the prosthesis, positive microbial culture, white blood cell count and proportion of polynuclear cells in joint fluid, serum CRP, and ESR were all elevated in the PJI group than in the AF group. The groups were similar in age, sex ratio and the location of prostheses (*P* >0.05).

**Table 1:** Clinical data of included patients

Characteristics	All patients (*n* = 37)	PJI (*n* = 24)	AF (*n* = 13)	*p*-value
Patient age, median (range)- years	65.38 ± 12.982	67.83 ± 11.38	60.85 ± 14.94	0.119
Gender, female, (no)%	17 (45.9)	9 (37.5)	8 (61.5)	0.161
Location, (no)%				0.823
Hip	19	12	7	
Knee	18	12	6	
Sinus	15	15	0	0.002
Antibiotics prior to surgery	16	15	1	0.002
Serum CRP, media (range)-mg/L	27.05 ± 39.74	39.31 ± 44.99	4.43 ± 1.76	0.001
ESR, media (range)-mm/h	52.11 ± 35.10	65.42 ± 34.81	27.54 ± 19.13	0.001
SF-WBC, media (range)- × 10^6^/L	25942.68 ± 62572.93	39722.13 ± 74594.79	503.69 ± 482.02	0.017
SF-PMN, media (range)-%	64.61 ± 21.37	76.22 ± 14.98	43.17 ± 13.11	0.001

*CRP* C-reactive protein, *ESR* erythrocyte sedimentation rate, *SF-WBC* synovial fluid white blood cell count, *SF-PMN* synovial fluid polymorphonuclear neutrophils

### Results of microbiological culture and mNGS

The pathogenic microorganisms detected by the culture and mNGS are listed in Table 2. Pathogenic microorganisms were isolated in 15 out of the 24 (62.5%) PJI cases using conventional culture, 12 cases of infections were caused by a single microorganism, and 3 were polymicrobial. Pathogenic microorganisms were isolated in 17 out of the 24 (70.8%) using sonication fluid culture. The 15 PJI cases with positive results in conventional and sonication fluid cultures had identical pathogenic microorganisms, whereas *Staphylococcus aureus* was isolated in the other 2 cases in sonication fluid cultures. Among a total of 10 microorganisms isolated by both culture methods, the predominant causative pathogens were coagulase-negative *Staphylococci* (*n* = 6, 33.3%), *Enterococcus faecalis* (n = 6, 33.3%) and *Staphylococcus aureus* (*n* = 4, 17.7%).

**Table 2:** Organisms of PJI detected by culture and mNGS

Microorganisms	No. of all detected micro-organisms	Conventional culture	Sonication culture	Sonication mNGS
*Staphylococcus aureus*	5	3	5	5
*Enterococcus faecalis*	6	4	4	5
*Staphylococcus haemolyticus*	1	1	1	1
*Helcococcus* *kunzii*	1	1	1	1
*Staphylococcus epidermidis*	4	4	4	4
*Finegoldia* *magna*	1	1	1	1
*Mycobacterium* *abscessus*	1	1	1	1
*Streptotoccus agalactiae*	1	1	1	1
*Pseudomonas aeruginosa*	1	1	1	1
*Escherichia coli*	1	1	1	1
*Parvimonas* *micra*	1	0	0	1
*Neisseria macacae*	1	0	0	1
*Candida parapsilosis*	1	0	0	1
*Mycoplasma hominis*	2	0	0	2
*Candida tropicalis*	1	0	0	1
*Enterobacter cloacae*	1	0	0	1
All	29	18	20	28

mNGS was used to analyze the sonication fluid collected from 24 cases in the PJI group, with an average number of 22577339 reads in each case (range, 14024588–29700868 reads). The average proportion of the human nucleic acid was 96.11% (range, 91.13–98.89%), and the average number of standardized stringently mapped reads number of genus (SDTMR) was 10215 (range, 47–118525 reads) in each case. The mNGS results of the 24 PJI cases were all positive when the threshold of RAG was set at 30%. The mNGS results were consistent at the genus and species levels with the results from 16 out of the 17 culture-positive cases (including conventional culture and sonication fluid culture). Nevertheless, only 1 case showed discordant result. While *Enterococcus faecalis* was isolated by the conventional and sonication fluid cultures, the mNGS result indicated that the pathogenic microorganism was *Enterobacter cloacae*. Pathogenic microorganisms were detected by mNGS in the sonication fluid of 7 culture-negative cases (case 2–8, Table 3), including *Parvimonasmicra *[1], *Mycoplasma hominis *[2], *Candida tropicalis* [1], *Neisseria macacae* [1], *Candida parapsilosis* [1] and *Staphylococcus epidermidis* [2]. Based on the mNGS results, 19 infections were caused by a single microorganism, and 5 were polymicrobial. Among a total of 10 microorganisms isolated by sonication fluid mNGS, the predominant causative pathogens were coagulase-negative *Staphylococci* (*n* = 8, 27.6%), *Staphylococcus aureus* (*n* = 5, 17.2%) and *Enterococcus faecalis* (*n* = 5, 17.2%). In the AF group, the results of conventional culture were all negative, while *Staphylococcus epidermidis* was detected by culture and mNGS in the sonication fluid in one case (Case 10, Table 3).

**Table 3:** Cases with discordant results between culture and mNGS

							Preoperative aspirate				
Case	Age (y)/sex	Diagnosis	Antibiotic Prior to surgery	ESR	CRP	Volume/feature	Conventional Culture	Sonication Fluid Culture	Sonication mNGS	WBC (cells/μL)	PMN%
1	90/M	THA infection	Not used	17	17.1	10 ml/bloody	Negative	*Staphylococcus aureus*	Staphylococcus aureus	7838	88.2
2	63/F	THA infection	Not used	29	2.83	5 ml/bloody	Negative	Negative	Parvimonasmicra	7160	83.9
3	49/M	THA infection	Not used	47	11.5	8 ml/yellow-sticky	Negative	Negative	Mycoplasma hominis	6380	63.8
4	79/F	TKA infection	Not used	16	2.8	8 ml/bloody	Negative	Negative	Candida tropicalis	820	47.9
5	66/F	TKA infection	Used	8.07	65	8 ml/yellow-sticky	Negative	Negative	Staphylococcus epidermidis	9144	75.9
6	63/M	THA infection	Used	58	40.2	9 ml/bloody	Negative	Negative	Staphylococcus epidermidis	6870	83.4
7	63/M	THA infection	Used	58	40.2	15 ml/bloody	Negative	Negative	Staphylococcus epidermidis& Enterococcus faecalis	6870	83.4
8	63/M	TKA infection	Used	65	12.3	15 ml/yellow-sticky	Negative	Negative	Neisseria macacae&Candida parapsilosis	32925	90.3
9	89/M	TKA infection	Used	120	90	34 ml/purulent	Negative	Negative	Mycoplasma hominis	26286	85.6
10	74/M	TKA infection	Used	16	5.62	6 ml/purulent	*Enterococcus faecalis*	*Enterococcus faecalis*	Enterobacter cloacae	1717	62.7
11	58/F	TKA aseptic failure	Not used	27	8.43	10 ml/Clean	Negative	*Staphylococcus epidermidis*	Staphylococcus epidermidis	186	26.8

### Analytical performance of sonication mNGS in the diagnosis of PJI

The performance of diagnostic tests is summarized in Table 4. The sonication fluid culture showed a sensitivity of 70.83% and a specificity of 92.31%, while the sensitivity of conventional culture (62.5%) was lower than that of the sonication fluid (*P* < 0.05). The sonication fluid mNGS exhibited a sensitivity of 100% and a specificity of 92.31%. The sensitivity of sonication fluid mNGS was significantly higher than those of the conventional and sonication fluid cultures (P < 0.05), while the specificity of sonication fluid mNGS (92.31%) was lower than that of the conventional culture, but the difference was not statistically significant (*P* > 0.05).

**Table 4:** Comparison of diagnostic efficiency between culture and mNGS

Positive Diagnostic Test	No. of patients, (*n* = 37)	PJI (*n* = 24)	AF (*n* = 13)	Sensitivity (%)	Specificity (%)	PPV (%)	NPV (%)	Accuracy (%)
Conventional Cultures	37	16/8	0/13	66.67	100	100	61.9	66.67
Sonication fluid cultures	37	17/7	1/12	70.83	92.31	94.44	63.16	63.14
Sonication fluid mNGS	37	24/0	1/12	100	92.31	96	100	92.31

*PPV* positive predictive rate, *NPV* negative predictive rate

### Discordant microbiological results

Case 1, a 90-year-old male had undergone a right total hip arthroplasty (THA) five years ago, and a sinus tract was present at the incision 8 months ago. At admission, his CRP level was 17.1 mg/L and ESR was 17 mm/h. The white blood cell count of synovial fluid was 7,838 × 10^6^/L with 81.9% being neutrophils. The results of the conventional microbial culture were all negative. However, *Staphylococcus aureus* was identified in the sonication fluid by culture, the diagnosis of PJI was definitely made according to MSIS criteria. mNGS analysis of the genomic DNA from sonication fluid also yielded 26 reads for *Staphylococcus aureus*.

Although the conventional culture and sonication fluid culture results were all negative in Cases 2–9, the 8 cases were all diagnosed as having PJI according to the MSIS criteria. Case 2 was a 65 year-old female who underwent a left THA due to developmental dysplasia of left hip. Three years ago, she found a gradually enlarging mass in her left thigh and the MRI showed effusion around the femur and a heterogeneous mass connected to the femur defect via an underlying tract and the analysis of synovial fluid revealed that the white blood cell count was 42,478 × 10^6^/L with 92.3% neutrophils. The mNGS results of Case 2 suggested that the pathogenic microorganism was *Parvimonasmicra* (23887 reads). Case 3 in PJI group was a 49-year-old male who underwent a right THA due to femoral neck fracture 8 years ago. One year ago, he felt intermittent right hip pain that was exacerbated by movement and CRP was 11.5 mg/L, ESR was 47 mm/h and the analysis of synovial fluid revealed that the white blood cell count was 6380 × 10^6^/L with 63.8% being neutrophils. The mNGS results indicated *Mycoplasma hominis* infection (3136 reads) while conventional culture and sonication fluid culture showed negative results. Case 4 in PJI group was a 79 year-old female who underwent a right total knee arthroplasty (TKA) due to knee osteoarthritis one year ago. After the surgery, she suffered from mild and intermittent left knee pain that was exacerbated by movement. A sinus tract was present at the incision 2 months ago and the X-ray showed prosthesis looseness in the tibial side, however, her CRP (2.8 mg/L), ESR (16 mm/h), synovial WBC (820 × 10^6^/L) and neutrophil percentage (47.9%) were all normal. Meanwhile, the results of conventional culture and sonication fluid culture were negative and the mNGS detected *Candida tropicalis* (2240 reads) in the sonication fluid. Case 5 was a 66 years-old female who was diagnosed as having PJI because of the presence of the sinus tract and her CRP was 65 mg/L, ESR was 8.07 mm/h and the analysis of synovial fluid revealed that the white blood cell count was 9,144 × 10^6^/L with 75.9% being neutrophils at this point. This patient had received antibiotic treatment in another hospital before sampling, thus, the results of conventional culture and sonication fluid culture were all negative. However, *Staphylococcus epidermidis* (3051 reads) were detected in sonication fluid by mNGS. Case 6 was a 63 year-old male who was assigned into PJI group because his CRP (40.2 mg/L), ESR (58 mm/h), synovial WBC (6870 × 10^6^/L) and neutrophil percentage (83.5%) were all markedly elevated. This patient also had received antibiotic treatment in another hospital before sampling, thus, the results of conventional culture and sonication fluid culture were negative. However, *Staphylococcus epidermidis* (38534 reads) was detected in sonication fluid by mNGS. Case 7 was a 63 year-old male who underwent right THA 10 years ago because of osteonecrosis of the femoral head (NOFH). A sinus was present at the incision 3 years ago and he was treated with debridement surgery and antibiotics several times in another hospital. His CRP (40.2 mg/L), ESR (58 mm/h), synovial WBC (6780 × 10^6^/L) and neutrophil percentage (83.4%) were all markedly elevated when re-admitted. Case 8 was a 63 year-old male with a repeated progressive swelling and pain in the right knee after TKA for 1.5 years and at this point, the patient’s CRP (12.3 mg/L), ESR (65 mm/h), synovial WBC (32925 × 10^6^/L) and neutrophil percentage (90.3%) were all markedly elevated. The results of conventional culture and sonication culture were all negative. mNGS detected *Neisseria macacae* (101 reads) and *Candida parapsilosis* (1022 reads) in sonication fluid. Case 9 was a 89 year-old male who underwent bilateral unicompartmental knee arthroplasty (UKA). Five weeks after the surgery, the right knee was still swollen and pus oozed out from the incision. At this point, his CRP was 90 mg/L, ESR was 120 mm/h and the analysis of synovial fluid revealed that the white blood cell count was 26286 × 10^6^/L with 85.6% being neutrophils, however, the results of conventional culture and sonication fluid culture were negative and *Mycoplasma hominis* (2491 reads) was detected in sonication fluid by mNGS. Case 10 was a 74-year-old male who had undergone a left TKA 2 months before re-admission and a sinus tract was present at the incision. At admission, his CRP level was 5.62 mg/L and ESR was 16 mm/h. The white blood cell count of synovial fluid was 17179 × 10^6^/L with 62.7% being neutrophils. *Enterobacter cloacae* was identified with 919 reads by mNGS, whereas *Enterococcus faecalis* was isolated by conventional and sonication fluid culture. Case 11 was a 58-year-old female who had undergone a right TKA 7 years ago, and was diagnosed with aseptic failure according to the MSIS criteria. When admitted, her CRP level was 8.43 mg/L and ESR was 27 mm/h. The white blood cell count of synovial fluid was 186 × 10^6^/L with 26.8% neutrophils. Although the results of conventional culture were negative, the results of sonication fluid culture and mNGS showed the presence of *Staphylococcus epidermidis*.

## Discussion

mNGS can sequence all nucleic acid fragments in a clinical sample, enabling the use of bioinformatic methods to obtain microbial sequences and species information. Compared with the PCR-based diagnostic technologies, mNGS has many advantages, such as the capability of simultaneously identifying bacteria and fungi in a single assay and obtaining quantified abundances of microorganisms for distinguishing causative pathogens [23]. As early as in 2014, Chiu et al. reported a successful mNGS-based detection of *Leptospira *from the cerebrospinal fluid of a patient with long-term unexplained fever, indicating that mNGS is being gradually implemented in the field of clinical microbial diagnosis [24]. Moreover, owing to the substantial cost reduction, mNGS has been increasingly applied to the diagnosis of orthopaedic infection [18].

Sonication can effectively destroy the biofilm formed by bacteria on the surface of prosthesis, and can thus increase the amount of bacteria in the samples. Trampuz et al. first reported the use of sonication to treat 120 joint prosthesis, and found that the detection rate of bacteria was improved [25]. However, in this cohort study there was no statistically significant difference between the detection rate of sonication fluid culture and that of conventional culture. One explanation was that sonication increases the penetration of antibiotics into bacterial cell membranes. Another possible reason was that sonication cannot reduce the occurrence of false-negative result caused by the fastidious microorganisms. Thus, we introduced the mNGS to identify the fastidious microorganisms and overcome problem with the low activity of microorganisms caused by the sonication and antibiotics. The sonication fluid mNGS exhibited a sensitivity of 100% and a specificity of 92.31%. The sensitivity of sonication fluid mNGS was significantly higher than that of the conventional and sonication fluid cultures (*P* < 0.05), and the specificity of sonication fluid mNGS (92.31%) was lower than that of the conventional culture, although the difference was not statistically significant (*P* > 0.05). The mNGS results were consistent with the those of the culture-based methods at both genus and species levels in 16 out of the 17 (94.12%) culture-positive cases. Meanwhile, mNGS could detect pathogenic microorganisms in the sonication fluid of all 7 culture-negative PJI cases, and two of them were caused by multiple microorganisms. The detection rate of pathogenic microorganisms in the PJI group of this study was higher than that reported by Ivy et al., who analyzed the synovial fluid of PJI cases with mNGS [17]. The difference is possibly due to the use of sonication fluid for mNGS in the present work. The biofilm attached to the surface of the prosthesis was destroyed by sonication, thereby increasing the load of bacterial DNA in the sample, in spite of decreased bacterial activity after sonication.

In our study, Case 1 underwent right THA and a sinus was present at the incision 8 months ago. Conventional culture showed negative results, while *Staphylococcus aureus* was isolated in the sonication fluid and the mNGS result was consistent with that of sonication fluid culture. This phenomenon may be due to the fact that the biofilm was disrupted by sonication, more microorganisms were released into the sample, and *Staphylococcus aureus* is a kind of common pathogenic microorganisms that can be isolated by culture in the clinical laboratory. Therefore, the results of sonication fluid culture and mNGS were consistent. The mNGS results of Case 2 suggested that the pathogenic microorganism was *Parvimonasmicra*, which belongs to Gram-positive obligate anaerobic cocci and requires highly stringent culture conditions. Accordingly, its detection rate of culture is low. Thus, we have to develop an antibiotic regimen according to the result of mNGS. The patient underwent a 10-week antibiotic treatment (piperacillin/tazobactam 4.5 g q8h for 2 weeks and oral amoxicillin 0.5 q8h for 8 weeks). Finally, the infection was effectively controlled [26]. In Case 3, mNGS results indicated *Mycoplasma hominis* infection. *Mycoplasma hominis* is a rare pathogenic microorganism and is difficult to culture without highly specific conditions [27]. Consequently, the results of microbial cultures were all negative in this case. Case 4 was diagnosed as having PJI according to MSIS major criteria. Meanwhile, the results of conventional culture and sonication fluid culture were negative and the mNGS detected *Candida tropicalis* in the sonication fluid. These 3 cases suggested that mNGS is able toto detect some fastidious microorganisms in sonication fluid. Case 5 were diagnosed as having PJI because of the presence of the sinus tract and Case 6 were assigned into PJI group according to MSIS minor criteria. Both patients also had received antibiotic treatment in another hospital before sampling, thus, the results of conventional culture and sonication fluid culture were all negative. However, *Staphylococcus epidermidis* were detected in sonication fluid of both patients by mNGS. In Case 7, the results of conventional culture and sonication fluid culture were negative, however, *Staphylococcus epidermidis* and *Enterococcus faecalis* were detected in sonication fluid by mNGS. These 3 cases suggested that the mNGS results of sonication fluid are minimally affected by antibiotic treatment. Case 8 was diagnosed as PJI according to the MSIS minor criteria. The results of conventional culture and sonication culture were all negative. mNGS detected *Neisseria macacae* and *Candida parapsilosis* in sonication fluid. In Case 9, the results of conventional culture and sonication fluid culture were negative and *Mycoplasma hominis* was detected in sonication fluid by mNGS. In the above 2 cases, we tested different culture media, sonicated the removed prosthesis, and used prolonged culture time (> 14 days), but still could not detect the pathogenic microorganisms by culture. The negative-culture results may be attributed to the use of antibiotics and characteristics of the fastidious microorganisms. These 3 cases suggested that mNGS can detect some fastidious microorganisms in sonication fluid under the premise, even the patients had received antibiotics before sampling. The results of Case 2–9 indicated that the combination of sonication and mNGS can effectively decrease the false-negative results due to some rare and fastidious pathogenic microorganisms and the use of antibiotics. Meanwhile, mNGS of the sonication fluid can identify the pathogenic microorganisms and guide clinical medication within 72 h. Finally, all 7 patients were treated by surgery and antibiotics according to the mNGS results, no signs of recurrent infection were found during the postoperative follow-up. In Case 10, culture-based result (*Enterococcus faecalis*) was discordant with that of mNGS (*Enterobacter cloacae*). The authors propose that the infection was possibly caused by multiple microorganisms (not *Enterococcus faecalis *alone), which may be due to the poor incision healing. The control of infection was ineffective when it was treated by Vancomycin alone. We improved the antibiotic regimen by adding Meropenem. Thereafter, the incision of the patient successfully healed, and the infection was controlled. The failed detection of *Enterococcus faecalis* by mNGS can be explained that the number of reads belonging to *Enterococcus faecalis* or the degree of genome coverage was insufficient to allow BLAST alignment with the reference genomes in the database. The results of sonication fluid culture and mNGS were indicative of *Staphylococcus epidermidis* infection in Case 11 in the AF group. Meanwhile, the results of conventional culture were negative. One explanation is that *Staphylococcus epidermidis* is a common bacterial species, and may become the contaminating bacteria introduced during the sonication process. Another explanation, the widely applied MSIS criteria were used as the “golden standard” in this study, which included the conventional culture results as the major criteria. This case was defined as AF by negative conventional culture results, and if it was a false negative, it might be a categorization errors. Therefore, in this case, it has yet to be determined based on subsequent clinical symptoms whether hidden infection is present. At present, the patients were only followed up for 3 months, and the CRP and ESR are temporarily at normal levels. However, the possibility of infection is not yet excluded, and further follow-up is needed.

This study has the following limitations: [1] There were only 24 patients in the PJI group and 13 patients in the AF group due to the low total incidence of PJI. In addition, this work was a single-center study, and further multi-center research is necessary. [2] In this study, the genome coverage was too low to identify the resistance-related genes of pathogenic microorganisms. Therefore, it is difficult to predict the drug resistance of these microorganisms. In the future work, it is desirable to perform targeted detection of the drug resistance genes or increase the depth of sequencing and genome coverage of pathogens to improve the detection rate of drug resistance genes. [3] Although the MSIS criteria has been widely used, it is still impossible to avoid the offset caused by categorization errors in some cases. [4] In comparison with the traditional culture methods, extensive application of mNGS is limited by the expensive equipment and the high operational costs. As a result, it is difficult for many hospitals and laboratories to be equipped with mNGS. However, the BGI-500 sequencing platform and automated bioinformatic analysis process used in this study can cost-effectively complete the detection and interpretation of the results within 48 h after receiving the samples.

## Conclusion

We demonstrated that mNGS of the sonication fluid can improve the detection rate of pathogenic microorganisms and provide valuable information for the diagnosis of PJI, and the mNGS results were highly consistent with the those of culture. In addition, mNGS can effectively identify pathogenic microorganisms in culture-negative PJIs cases, especially in the cases treated with antibiotics before surgery or those having rare or fastidious microorganisms. Therefore, this method can potentially help to guide the clinical use of antibiotics.

## Abbreviations

AF: Aseptic failure
CR: Coverage rate
CRP: C-reactive protein
ESR: Erythrocyte sediment rate
mNGS: Metagenomic next-generation sequencing
MSIS: The Musculoskeletal Infection Society
MTCP: *Mycobacterium tuberculosis* complex
NGS: Next-generation sequencing
NPV: Negative predictive rate
PCR: Polymerase chain reaction
PJI: Periprosthetic joint infection
PPV: Positive predictive rate
RAG: The relative abundance at the genus level
SDTMR: Standardized stringently mapped reads number of genus
SMRN: The number of reads stringently mapped to pathogen species
